# Tongue muscle strength and resting posture across sagittal skeletal patterns in adolescents: a cross-sectional study using mediation analysis and decision tree

**DOI:** 10.1186/s12903-026-08935-x

**Published:** 2026-06-22

**Authors:** Jiahui Ye, Yidan Guo, Qiang Sun, Jia Teng, Liuyuan Wang, Yanning Ma

**Affiliations:** Shanxi Medical University School and Hospital of Stomatology, Shanxi Province Key Laboratory of Oral Diseases Prevention and New Materials, Taiyuan, 030001 China

**Keywords:** Tongue, Sagittal skeletal patterns, Mediation analysis, Decision tree

## Abstract

**Background:**

Tongue function significantly influences craniofacial development, yet the morpho-functional characteristics of the tongue across different sagittal skeletal patterns remain unclear. This study aimed to quantitatively evaluate tongue muscle strength and resting posture in adolescents, delineate their biomechanical pathways, and construct data-driven diagnostic models for sagittal skeletal patterns.

**Methods:**

A cross-sectional study was conducted on 182 adolescents (11–17 years) stratified into Class I, II, and III sagittal patterns based on the ANB angle. Maximum active tongue pressure (MTP) was measured using a digital dynamometer. Resting posture, including the Tongue Tip Index (TTI), Tongue Tip Distance (TTD), and Tongue Dorsum Distance (TDD), was evaluated using lateral cephalograms. Data were analyzed using multivariable linear regression analysis, mediation analysis, and a Chi-squared Automatic Interaction Detection (CHAID) algorithm.

**Results:**

The Class III cohort exhibited significantly reduced MTP (median: 1.65 kg) compared to Class I and II subjects (~ 2.20 kg; *P* < 0.001). Mediation analysis identified a partial mediation relationship: while MTP demonstrated a significant direct statistical association with the ANB angle, 32.4% of the total association was indirectly mediated through TTI. The CHAID model identified reduced MTP (≤ 1.85 kg) as the primary root node associated with Class III risk. TTI (≤ 2.57%) served as a critical secondary stratifier of Class III risk.

**Conclusions:**

In adolescents, skeletal Class III malocclusion is associated with reduced tongue pressure and a lowered resting posture. Tongue muscle force demonstrates both a direct statistical association with the sagittal skeletal base and an indirect association mediated by the maintenance of an appropriate tongue tip position. The diagnostic model highlights reduced MTP as the primary risk indicator for Class III patterns, which is further stratified by TTI.

## Introduction

 Malocclusion etiology is multifactorial, involving both genetic and environmental factors. Notably, functional soft tissues exert a major influence on dentofacial development [1, 2]. According to Moss’s Functional Matrix Hypothesis, skeletal units act as secondary structures adapting to the biomechanical demands of the surrounding soft tissue [3]. Because the maxillofacial complex undergoes rapid expansion during the adolescent growth spurt, it becomes highly susceptible to altered soft tissue forces [4]. Therefore, evaluating these functional parameters during this period is essential for individualizing orthodontic treatment and minimizing relapse [5].

Within the orofacial system, the tongue exerts a continuous influence on dentofacial morphology [6]. As a muscular hydrostat composed of intrinsic and extrinsic muscles, the tongue exhibits significant morphologic adaptability [7]. Under physiologic conditions, the resting tongue elevates against the hard palate. The apex rests posterior to the incisive papilla, while the lateral borders engage the maxillary posterior alveolar ridges without pressing against the anterior dentition [6]. This palatal contact generates negative intraoral pressure, providing transverse support to the developing maxilla [8]. Conversely, a low resting tongue posture—characterized by the loss of palatal contact—deprives the maxilla of this internal support, which may impede proper sagittal development. Moreover, a low tongue posture frequently coincides with functional mandibular protrusion or accelerated mandibular body growth, contributing to the pathogenesis of skeletal Class III malocclusion [9].

While static resting posture is associated with sagittal skeletal patterns, maintaining an appropriate resting posture theoretically relies on active baseline muscle tone. As a muscular hydrostat devoid of rigid internal support, the tongue utilizes its baseline muscular tension to regulate its three-dimensional morphology [7]. However, the existing literature typically separates static cephalometric evaluations of tongue position from dynamic functional assessments [10–13]. To date, the concurrent quantification of tongue muscle strength and resting posture within a single cohort to explore their intrinsic relationships and guide clinical diagnosis remains unexplored.

To address these limitations, the present study investigated the association between tongue muscle strength and resting tongue posture. Subsequently, mediation analysis and the Chi-squared Automatic Interaction Detection (CHAID) algorithm were incorporated. The mediation analysis aimed to map the pathway through which reduced tongue pressure affects postural collapse and Class III skeletal development. Concurrently, the CHAID model assessed the diagnostic weight of these indicators for early risk stratification.

Ultimately, this study aims to evaluate tongue muscle strength and resting posture across various sagittal skeletal patterns. By utilizing mediation and decision tree analyses, we seek to provide quantitative evidence regarding the role of tongue musculature, particularly in the pathogenesis of skeletal Class III malocclusion, thereby offering reliable clinical parameters for early screening and interceptive therapy.

## Materials and methods

### Study population

This study adhered to the Declaration of Helsinki (https://www.wma.net/policies-post/wma-declaration-of-helsinki/). This cross-sectional study was approved by the Medical Ethics Committee of the Shanxi Medical University School and Hospital of Stomatology (Approval No. 2026SLL006). Written informed consent was obtained from all participants and their legal guardians. The sample comprised patients who sought orthodontic treatment between January 2026 and March 2026. The inclusion criteria were defined as follows: (1) chronological age ranging from 11 to 17 years; (2) presence of a fully erupted permanent dentition (excluding third molars); (3) presentation for initial comprehensive orthodontic evaluation; and (4) availability of standardized, pre-treatment lateral cephalograms acquired prior to any clinical intervention. Patients were excluded based on the following criteria: (1) poor radiographic quality precluding landmark identification; (2) a history of orthodontic or myofunctional therapy; (3) prior adenoidectomy or tonsillectomy; (4) craniofacial anomalies (e.g., cleft lip and palate) or a history of maxillofacial trauma or surgery; (5) absence of ≥ 2 consecutive anterior teeth in either arch; (6) radiographic evidence of altered dorsal tongue mucosa (wavy or curled); (7) tongue morphological anomalies (macroglossia or microglossia) or prior tongue surgery; and (8) deleterious oral habits (e.g., predominant mouth breathing, atypical swallowing patterns, or thumb sucking).

An a priori sample size calculation was performed using G*Power software (Version 3.1.9.7, Heinrich-Heine-Universität, Düsseldorf, Germany). Assuming a medium effect size (Cohen’s *f* = 0.25) for the primary functional outcome (maximum tongue pressure) among the three sagittal skeletal classes, an alpha level of 0.05, and a statistical power of 80%, a minimum sample size of 159 subjects was required. Furthermore, the recruited cohort of 182 participants satisfied the heuristic sample size recommendations for mediation analysis and provided sufficient case density for the recursive partitioning and cross-validation procedures of the CHAID model [14, 15]. Consequently, the sample size was considered adequate for both primary inferential testing and secondary multivariate modeling.

### Cephalometric analysis

**Fig. 1:**
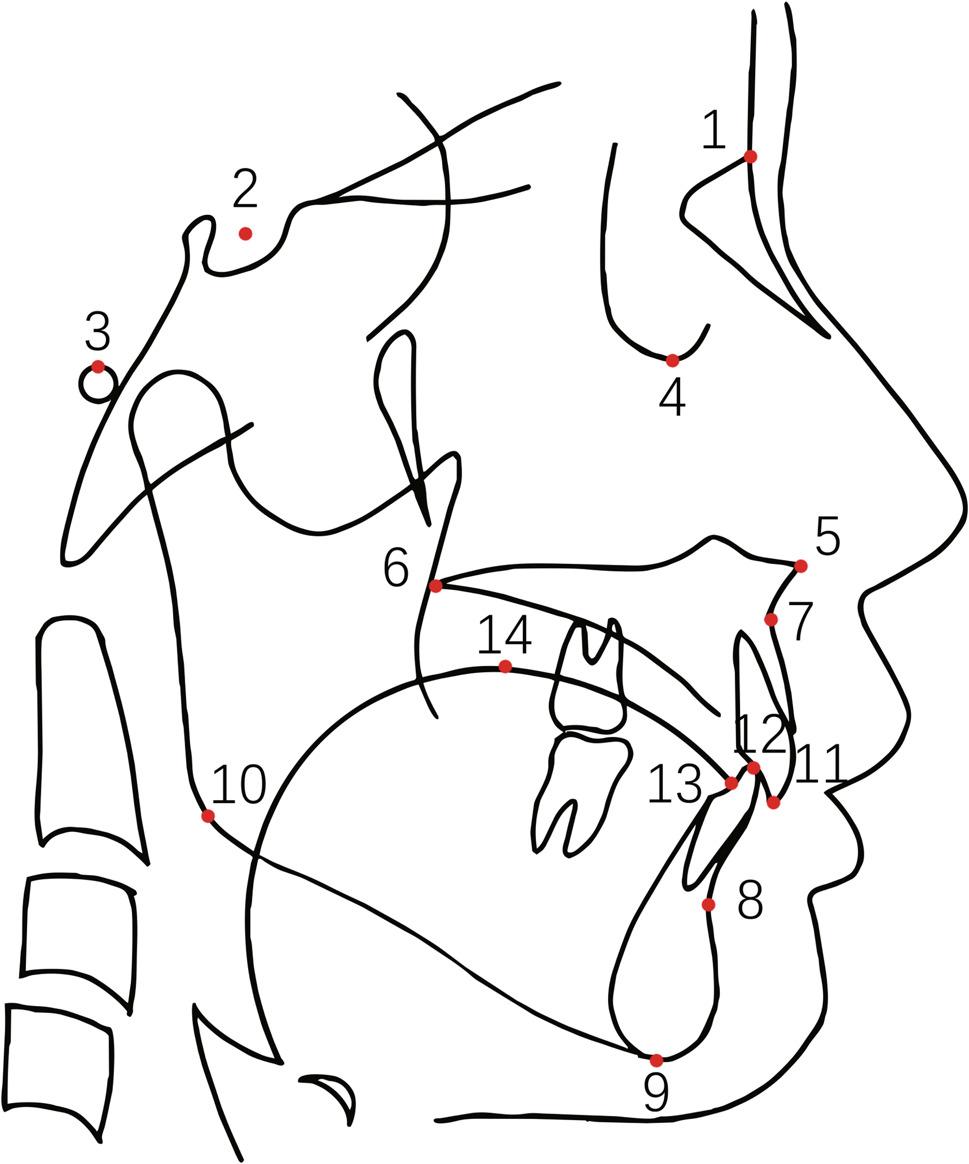
Reference points and lines on lateral cephalograms. 1, N; 2, S; 3, P; 4, Or; 5, ANS; 6, PNS; 7, A; 8, B; 9, Me; 10, Go; 11, U1; 12, L1; 13, TT; 14, TD

**Fig. 2:**
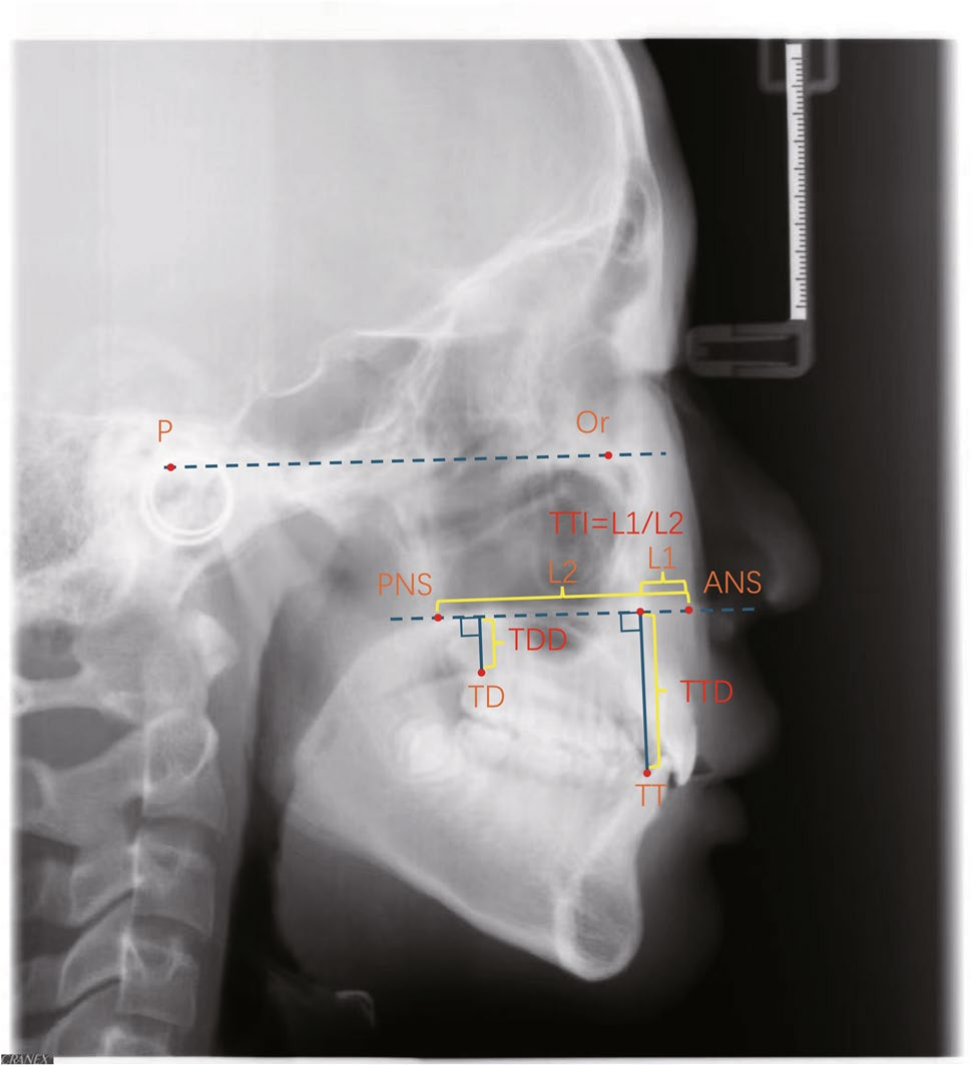
Cephalometric landmarks and measurements for the evaluation of resting tongue posture. The upper dashed line represents the Frankfort horizontal plane (Porion to Orbitale). The lower dashed line represents the palatal plane (ANS–PNS). TTD and TDD represent the perpendicular vertical distances from the tongue tip (TT) and the highest point of the tongue dorsum (TD) to the reference plane, respectively. The Tongue Tip Index (TTI) is calculated as the ratio of L1 (the sagittal distance from ANS to the perpendicular projection of TT) to L2 (the total reference length of the palate)

Lateral cephalograms were acquired following a standardized institutional protocol. To establish a reproducible natural head position, patients were instructed to stand upright and look into an eye-level mirror, with the Frankfort Horizontal (FH) plane maintained parallel to the floor (± 5° deviation limit) [16]. Prior to exposure, resting tongue posture was standardized: patients achieved maximum intercuspation, performed a swallowing reflex, and maintained a relaxed tongue posture with competent lips. Digital tracing and cephalometric measurements were performed using FusionOne software (Version 2.0.1.4444; LargeV, Beijing, China). The evaluated cephalometric landmarks and measurements are detailed in Table 1, with reference points and planes illustrated in Figs. 1 and 2.

**Table 1:** Cephalometric landmarks and measurements

Landmarks/Value	Definition
Sella, S	The midpoint of the sella turcica
Nasion, N	The most anterior point of the nasofrontal suture in the median sagittal plane
Porion, P	The most superior point of the external auditory meatus outline
Orbitale, Or	The deepest point on the infraorbital margin
Anterior nasal spine, ANS	Anterior tip of the median palate
Posterior nasal spine, PNS	The most posterior point of the posterior nasal spine on the palatine bone
Subspinale, A	The most posterior point on the anterior contour of the upper alveolar process
Supramental, B	The most posterior point on the anterior contour of the lower alveolar process
Menton, Me	The most inferior point on the outer inferior margin of the mandible
Gonion, Go	The most posterior-inferior point on the outline of the mandible angle
Upper incisor, U1	The most anterior-inferior point of upper incisors
Lower incisor, L1	The most anterior-superior point of lower incisors
Tongue tip, TT	The most inferior point of tongue tip
Dorsum of tongue, TD	The closest point on the tongue dorsal mucosa from palatal plane (ANS-PNS)
SNA	Sella-nasion-subspinale
SNB	Sella-nasion-supramental
ANB	Subspinale-nasion-supramental
FMA	The angle between the FH plane and the mandibular plane (Go-Me line)
Tongue Position Measurements	
Tongue tip distance, TTD	The linear distance between TT and palatal plane, indicating the vertical position of the tongue tip
Tongue dorsum distance, TDD	The linear distance from Td to the palatal plane, indicating the vertical position of the tongue
Tongue tip index, TTI	The ratio of the distance from TT’ to ANS to the distance of the ANS to PNS. TT’: the perpendicular projection of TT onto the palatal plane TT’ (the perpendicular projection of TT onto the palatal plane TT’ (the perpendicular projection of TT onto the palatal plane \(TT’ is the perpendicular projection of TT onto the palatal plane), indicating the sagittal position of the point TT. Expressed as TTI = TT’-ANS / ANS-PNS

To quantitatively assess the spatial orientation of the resting tongue, three specific morphometric indicators were defined relative to the palatal plane (ANS-PNS). The Tongue Tip Index (TTI) evaluated the sagittal position of the tongue tip (TT). It was calculated as the ratio of the distance from the anterior nasal spine (ANS) to TT’ (the perpendicular projection of TT onto the palatal plane) to the total palatal length (ANS-PNS). The Tongue Tip Distance (TTD) quantified the vertical position of the tongue tip, measured as the perpendicular linear distance from TT to the palatal plane. Similarly, the Tongue Dorsum Distance (TDD) assessed the vertical position of the tongue body, measured as the perpendicular linear distance from the highest point of the tongue dorsum (TD) to the palatal plane.

All cephalometric measurements were performed by a trained orthodontic graduate student who received standardized instruction in the use of the measurement software prior to the start of data collection. To evaluate intra-examiner reliability, 20 randomly selected lateral cephalograms were re-analyzed by the same examiner following a four-week interval. Measurement consistency was assessed using the intraclass correlation coefficient (ICC). All angular and linear parameters yielded ICC values exceeding 0.85. Consequently, the initial recordings were utilized for all subsequent statistical analyses.

### Tongue muscle strength assessment

**Fig. 3:**
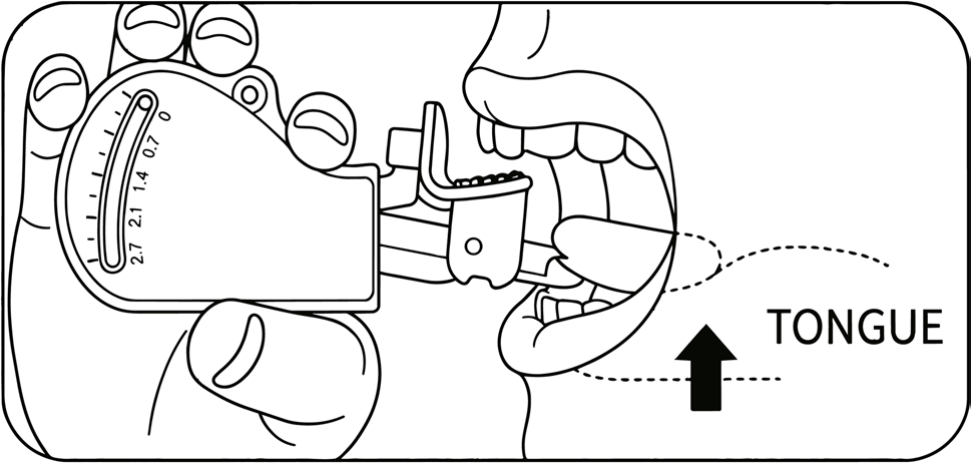
Schematic of maximum dynamic tongue pressure (MTP) measurement

Maximum tongue muscle force was quantified using a tongue dynamometer (LinkedSmile, Wuxi, China). The dynamometer spoon was positioned on the dorsal surface of the tongue, with the upper central incisors resting on the superior concave surface of the device. With a slightly open mouth, patients were instructed to apply maximum superior force against the spoon until peak resistance was reached. (Fig. 3) This peak value was recorded as the maximum active tongue pressure (MTP, in kg). To account for muscle fatigue and ensure measurement reliability, three consecutive recordings were obtained at 30-second intervals, and the mean value was calculated for statistical analysis.

### Statistical analysis

Statistical analyses were performed using IBM SPSS Statistics version 27.0 (IBM Corp., Armonk, NY, USA) and the PROCESS macro. Data normality was assessed via the Shapiro-Wilk test. Normally distributed variables were expressed as means ± standard deviations (SD), whereas non-normally distributed variables (e.g., MTP, TTI, ANB) were reported as medians with interquartile ranges (IQR). Categorical variables were presented as frequencies and percentages. For intergroup comparisons among the three sagittal skeletal classes (Class I, II, and III), one-way ANOVA was utilized for normally distributed continuous variables with homoscedasticity. The Kruskal-Wallis test was applied for non-normally distributed data, and the chi-square test was used for categorical variables.

A multiple linear regression analysis was conducted with the ANB angle as the dependent variable. Independent variables included tongue parameters (MTP, TTI, TDD, and TTD), while adjusting for dental compensations (L1-MP and U1-SN). The unstandardized regression coefficient *(B)*, standard error (SE), and standardized regression coefficient *(β)* were calculated for each predictor. To ensure the validity of the regression model, multicollinearity among the independent variables was assessed using the Variance Inflation Factor (VIF). Furthermore, the independence of observations (autocorrelation of residuals) was evaluated using the Durbin-Watson statistic. The overall explanatory power of the model was represented by the adjusted *R²* value.

Mediation analysis, utilizing the SPSS PROCESS macro, evaluated the pathways linking MTP (independent variable) to the ANB angle (dependent variable), with TTI serving as mediators. Direct and indirect effects were calculated using a bias-corrected bootstrapping method (5,000 resamples). Statistical significance for the mediating effect was determined if the 95% confidence interval (CI) excluded zero [17].

A Chi-squared Automatic Interaction Detection (CHAID) decision tree model was constructed for the risk stratification of skeletal Class III malocclusion (dependent variable) based on tongue indicators, including MTP and TTI (independent variables). Recursive partitioning was determined by chi-square significance testing, selecting the predictor yielding the lowest *P*-value at each node. The significance level for splitting nodes and merging categories was set at an alpha of 0.05, and the Bonferroni adjustment was applied to all *P*-values to correct for multiple comparisons. The model employed a 10-fold cross-validation approach. Predefined stopping rules included a maximum tree depth of 5, a minimum of 40 cases for parent node splitting, and a minimum of 10 cases per terminal node [18]. To evaluate the predictive performance and clinical utility of the established CHAID model, a Receiver Operating Characteristic (ROC) curve analysis was performed, and the overall discriminatory ability was quantified using the Area Under the Curve (AUC). Furthermore, diagnostic metrics including sensitivity, specificity, positive predictive value (PPV), and negative predictive value (NPV) were derived from the classification matrix. The 95% confidence intervals (CIs) for sensitivity and specificity were calculated using the Wilson score method to ensure the robustness of the precision estimates.

## Results

The final sample comprised 182 growing adolescents, stratified into three sagittal skeletal groups based on the ANB angle and molar relationships: Class I (*n* = 77), Class II (*n* = 53), and Class III (*n* = 52).

### Baseline Demographics and Bivariate Correlations

**Fig. 4:**
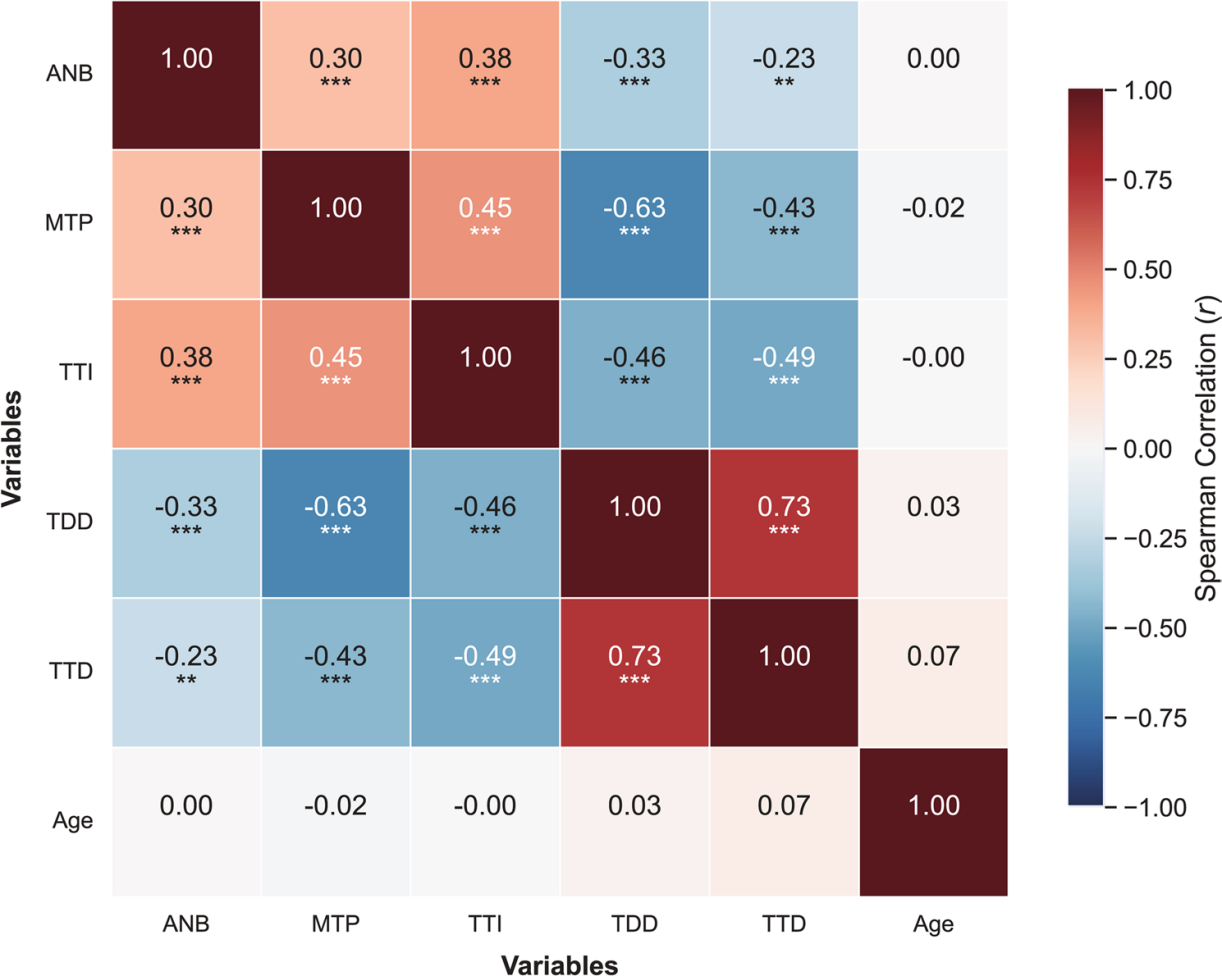
Spearman correlation matrix of morpho-functional and demographic variables. The heatmap illustrates the bivariate relationships among ANB, MTP, TTI, TDD, TTD and age. The color gradient reflects the magnitude and direction of the Spearman correlation coefficient *(r)*, ranging from dark blue (strong negative correlation, -1.00) to dark red (strong positive correlation, 1.00). Values within the individual cells represent the exact *r* values. Asterisks indicate the level of statistical significance. ***P < 0.001

Demographic characteristics were comparable across the three skeletal cohorts. Mean ages did not differ significantly (Class I: 13.69 ± 1.97; Class II: 13.36 ± 1.88; Class III: 13.25 ± 1.81 years; *P* = 0.390), and gender distribution remained balanced (*P* = 0.147). The primary grouping variable (ANB angle) demonstrated significant intergroup differences (Class I: 3.13°; Class II: 5.94°; Class III: -1.85°; *P* < 0.001) (Table 2). Spearman correlation coefficients were computed to evaluate the associations among craniofacial morphology, tongue muscle function, and resting posture (Fig. 4).

**Table 2:** Comparison of demographic and cephalometric variables

Variables	Class I (*n* = 77)	Class II (*n* = 53)	Class III (*n* = 52)	X² / F / H	*P* Value
Age/y	13.69 ± 1.97	13.36 ± 1.88	13.25 ± 1.81	0.948	0.390
Gender (Male/Female)	32/45	29/24	19/33	3.828	0.147
ANB/°	3.13 [2.23]	5.94 [1.27]	-1.85 [3.11]	158.61	< 0.001
MTP/kg	2.21 [0.50]	2.20 [0.70]	1.65 [0.60]	23.277	< 0.001
TTI/%	15.70 [11.33]	15.26 [9.32]	8.94 [10.67]	27.168	< 0.001
TTD/mm	20.30 [3.80]	22.33 [5.05]	24.68 [8.17]	20.839	< 0.001
TDD/mm	3.96 [2.47]	4.27 [4.79]	8.83 [6.80]	22.454	< 0.001
MPA/°	29.09 [5.40]	28.99 [11.00]	26.42 [10.5]	3.887	0.143
U1-SN/°	106.55 [11.71]	101.97 [12.03]	113.70 [11.10]	41.147	< 0.001
L1-MP/°	92.70 [9.84]	99.03 [11.59]	86.31 [12.94]	48.719	< 0.001

The ANB angle exhibited a significant positive correlation with MTP (*r* = 0.30, *P* < 0.001), indicating that skeletal Class III patterns frequently coincide with reduced tongue muscle strength. Morphologically, ANB correlated positively with TTI (*r* = 0.38) and negatively with both TDD and TTD (*r* = -0.33 and *r* = -0.23, respectively). Notably, MTP demonstrated a significant negative correlation with TDD (*r* = -0.63, *P* < 0.001) and a positive correlation with TTI (*r* = 0.45, *P* < 0.001), suggesting that muscular weakness is associated with a lowered, anteriorly displaced resting tongue posture (Fig. 4).

### Multivariable linear regression analysis

Multivariable linear regression was performed to evaluate the independent associations of tongue physiological and spatial parameters with the sagittal skeletal pattern (ANB angle). The model exhibited a robust fit, explaining 56.4% of the variance in the ANB angle (*R* ^*2*^ = 0.564, adjusted *R* ^*2*^ = 0.549). Collinearity diagnostics and the Durbin-Watson statistic (2.121) confirmed that the assumptions of non-multicollinearity (all VIFs < 4) and residual independence were successfully met.

After strictly controlling for dental compensation effects, lower incisor inclination (L1-MP: *β* = 0.465, *P* < 0.001) and upper incisor inclination (U1-SN: *β* = -0.389, *P* < 0.001) emerged as the strongest predictors. Among the tongue-related variables, MTP (*B* = 1.567, *P* = 0.001) and TTI (*B* = 0.078, *P* = 0.004) were identified as significant independent predictors, both demonstrating positive associations with the ANB angle. In contrast, TDD (*P* = 0.160) and TTD (*P* = 0.174) tongue position indices did not reach statistical significance in this multivariable model. (Table 3).

**Table 3:** Multivariable linear regression analysis predicting the ANB angle

Variables	Unstandardized B (SE)	Standardized β	t value	*P* value	VIF
(Constant)	-2.781 (2.784)	-	-0.999	0.319	-
Dental compensations					
L1-MP	0.173 (0.019)	0.465	8.953	< 0.001	1.083
U1-SN	-0.133 (0.018)	-0.389	-7.490	< 0.001	1.079
Tongue variables					
TDD	0.100 (0.071)	0.140	1.410	0.160	3.956
TTD	-0.055 (0.040)	-0.117	-1.366	0.174	2.966
TTI	0.078 (0.027)	0.173	2.905	0.004	1.423
MTP	1.567 (0.484)	0.220	3.238	0.001	1.845

### Mediating role of tongue posture on the muscle-bone relationship

**Fig. 5:**
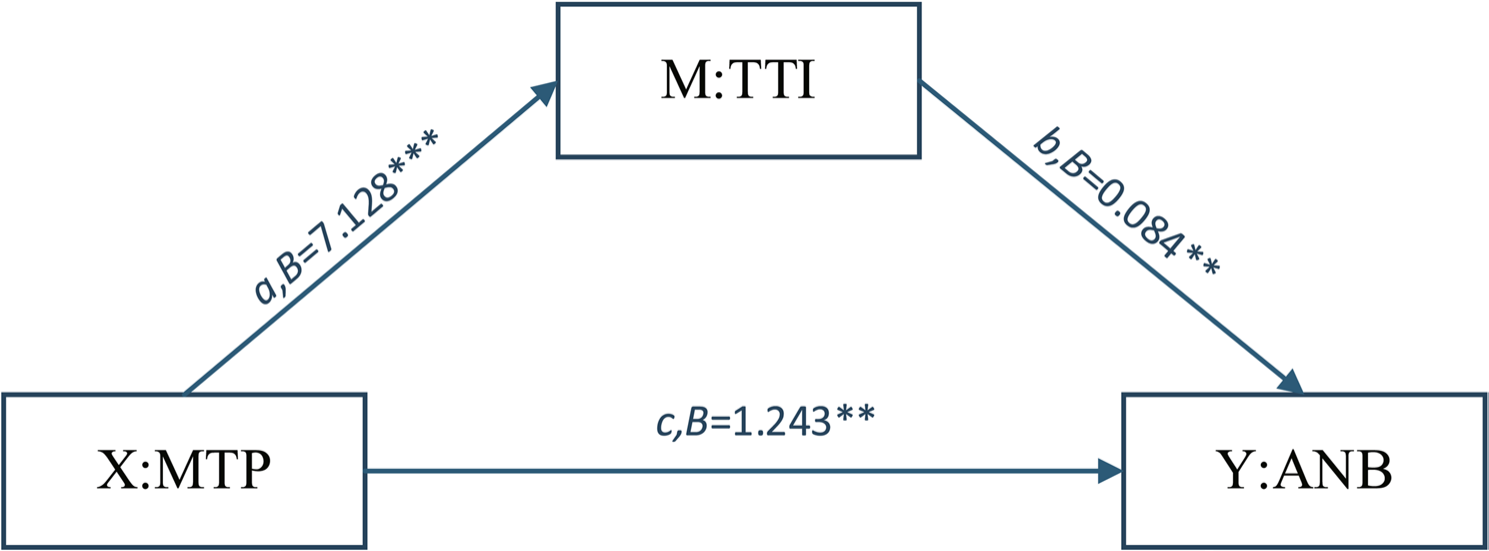
Path diagram of the mediation analysis. The model evaluates the direct and indirect pathways from MTP(X) to ANB(Y) through TTI(M). All displayed pathways are statistically significant. Unstandardized path coefficients *(B)* are presented for each link, illustrating a significant partial mediation effect of TTI on the relationship between tongue muscle force and the skeletal base. **P < 0.01, *** *P* < 0.001

Mediation analysis was applied to evaluate the pathways linking MTP and the sagittal skeletal pattern. We tested whether MTP (independent variable) directly impacts the ANB angle (dependent variable) or indirectly affects it via TTI (Fig. 5). The total effect of MTP on the ANB angle was significantly positive (B = 1.839, *P* < 0.001). When accounting for the mediator, the direct path coefficient from MTP to ANB remained statistically significant (*B* = 1.243, *P* = 0.003), indicating a partial mediation model.

In the mediation model, TTI demonstrated a statistically significant mediating effect. MTP positively predicted TTI (a = 7.128, *P* < 0.001), indicating an association between greater muscle force and a superiorly maintained resting tongue tip posture. In turn, TTI positively predicted the ANB angle (b = 0.084, *P* = 0.002). Consequently, the specific indirect pathway via TTI was confirmed to be significant (*B* = 0.596, 95% CI: 0.227–0.986), accounting for 32.4% of the total effect (Table 4).

**Table 4:** Mediation analysis: indirect effects of MTP on ANB

Effect Type	Path	Unstandardized B	SE	*P*	95% CI [LLCI, ULCI]
Total Effect (c)	MTP →ANB	1.839	0.428	< 0.001	[0.994, 2.683]
Direct Effect(c’)	MTP →ANB	1.243	0.406	0.003	[0.442, 2.044]
Indirect Effect (ab)	MTP →TTI →ANB	0.596	0.189	-	[0.227, 0.986]

### CHAID decision tree modeling for clinical risk stratification

A CHAID decision tree model was developed using MTP and TTI as core predictors to stratify the risk of skeletal Class III malocclusion. The algorithm identified MTP as the optimal root node, establishing a primary critical threshold at 1.85 kg. Subjects demonstrating robust tongue muscle force (MTP > 1.85 kg) were immediately categorized into a low-risk terminal node (Node 2), where only 13.8% of the cases presented with Class III malocclusion.

**Fig. 6:**
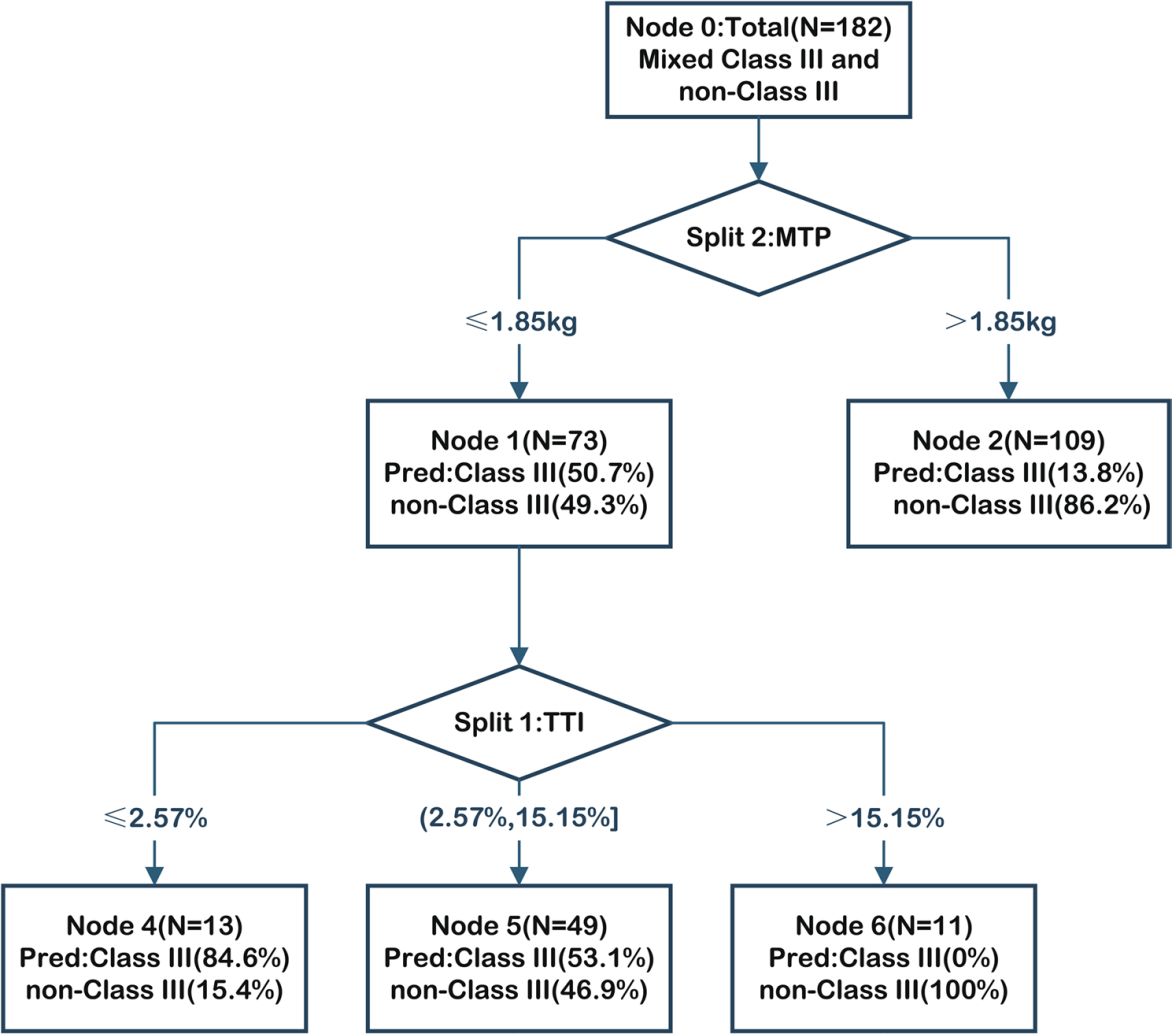
CHAID decision tree model for stratifying the risk of skeletal Class III malocclusion. The initial cohort (*N* = 182) was first divided by the primary root node using MTP, with a critical cut-off value of 1.85 kg. For the functionally susceptible subset (MTP ≤ 1.85 kg), TTI served as a secondary stratifier to further classify the risk based on spatial posture. The terminal nodes display the sample size (N) and the predicted probabilities of presenting with Class III or non-Class III (Class I and II) skeletal patterns for each subgroup

Conversely, for patients with diminished tongue muscle strength (MTP ≤ 1.85 kg, Node 1), the risk of Class III discrepancy notably increased to 50.7%. To further stratify this higher-risk cohort, the model recruited TTI as the secondary discriminatory node, dividing these patients into three distinct subgroups based on their resting tongue posture. Specifically, patients exhibiting a severely low resting posture (TTI ≤ 2.57%) were classified into the highest-risk terminal node (Node 3), where a striking 84.6% of cases were confirmed as Class III. On the other end of the spectrum, a high resting posture (TTI > 15.15%, Node 5) indicated a zero probability (0.0%) of a Class III discrepancy, effectively compensating for the low muscle strength. For patients falling into the intermediate TTI range (2.57% < TTI ≤ 15.15%, Node 4), the probability of Class III remained elevated at 53.1% (Fig. 6).

**Fig. 7:**
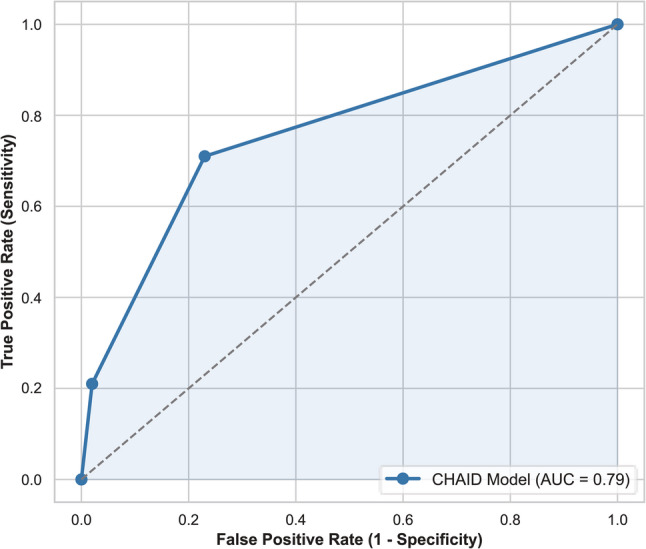
Receiver operating characteristic (ROC) curve analysis of the established CHAID decision tree model. The model evaluates the diagnostic performance of MTP and TTI in stratifying the risk of sagittal skeletal malocclusion. The area under the curve (AUC) is 0.79, demonstrating good overall discriminatory ability. The diagonal dashed line represents the reference line of a random classifier (AUC = 0.50)

The classification accuracy of the updated CHAID decision tree model was systematically evaluated. Since the target variable was binarized to isolate the Class III risk, the algorithm yielded an overall predictive accuracy of 78.0%. The model demonstrated robust predictive validity, correctly classifying 80.8% of the non-Class III subjects (specificity) and 71.2% of the actual Class III subjects (sensitivity) (Table 5). Furthermore, receiver operating characteristic (ROC) curve analysis revealed an area under the curve (AUC) of 0.79 (95% CI: 0.71–0.86) (Fig. 7).

**Table 5:** Diagnostic performance of the established CHAID decision tree model

Diagnostic Metric	Value (95% CI)
Cut-off value	> 0.50
AUC	0.79 (0.71–0.86)
Sensitivity (%)	71.2 (57.9–81.6)
Specificity (%)	80.8 (73.1–86.7)
Positive Predictive Value (%)	59.7
Negative Predictive Value (%)	87.5
Overall Accuracy (%)	78.0

## Discussion

Malocclusion is multifactorial, with soft tissue function contributing significantly to its pathogenesis [19]. This investigation evaluated the associations among tongue muscle function and resting posture across different sagittal skeletal patterns. By integrating morphological and functional parameters, this study aimed to explore the potential mechanisms through which soft tissue dynamics influence sagittal skeletal development. Although the influence of tongue function on craniofacial development is recognized, findings regarding the correlation between MTP and sagittal skeletal patterns (e.g., ANB angle) remain inconsistent [13, 20–24]. For example, Kurabeishi et al. [21] reported higher MTP in mixed-dentition children with skeletal Class III patterns compared to Class I and II subjects, attributing this to a relatively larger tongue volume. Conversely, Lee et al. [13] observed a weak negative correlation between anterior tongue pressure and the ANB angle in adults, with no significant association found for posterior pressure.

These discrepancies may stem from methodological differences in MTP quantification. Previous studies predominantly utilized balloon-type manometric devices (e.g., IOPI or JMS systems), which measure pressure via the deformation of an air-filled bulb [25]. However, the fixed intraoral volume of the bulb may introduce measurement bias [26]. In skeletal Class III patients, who frequently present with maxillary hypoplasia and relative macroglossia [27], the reduced oral cavity volume might lead to artificially elevated pressure readings. These values could reflect passive compression between the tongue and the constricted palate rather than active muscular contraction.

To minimize this volumetric confounding, this study utilized a dynamometer designed to quantify maximum dynamic pressure during active tongue elevation. Our findings indicate that patients with skeletal Class III malocclusion exhibit reduced tongue muscle force. While Class I and Class II subjects demonstrated comparable median MTP values (approximately 2.20 kg), the Class III cohort showed a significant reduction in active pressure generation (median MTP: 1.65 kg, *P* < 0.001).

Regarding spatial orientation, previous literature associates a low resting tongue posture with the Class III phenotype [10, 11, 28, 29]. The cephalometric data in this study align with these findings, demonstrating that skeletal Class III patients present with a lower dorsal contour and an anteriorly displaced tongue apex. Collectively, this suggests a combined phenotype of reduced muscular force and altered resting posture in Class III subjects. A limitation of prior research is the separate evaluation of strength and spatial positioning. By integrating these metrics, the present Spearman correlation analysis revealed significant associations between MTP and all evaluated postural variables (TDD, TTD, and TTI). These correlation patterns consistently suggest that reduced muscular strength is closely associated with a lowered resting tongue posture. This morpho-functional relationship aligns with the biomechanical principles of a ‘muscular hydrostat’. Without rigid cartilaginous or osseous support, the tongue relies entirely on its orthogonally arranged intrinsic and extrinsic muscle fibers to regulate its three-dimensional shape [30]. To maintain an elevated resting posture against the palate, the tongue must constantly counteract gravity. This requires continuous and coordinated muscular tension from the orthogonal network. Consequently, adequate resting tongue pressure serves as the fundamental prerequisite for sustaining the proper tongue posture. Another key feature of such hydrostatic structures is the principle of volume conservation, which means that any dimensional change in one axis forces compensatory adaptations in the remaining axes [31]. When diminished resting tongue pressure is insufficient to maintain this vertical elevation, the tongue descends (clinically observed as an increased TDD). Because the tongue maintains a constant volume, its mass must passively redistribute. Consequently, the tongue undergoes compensatory sagittal elongation. This elongation leads to a displaced tongue tip, which is reflected by a decreased TTI. This study quantified dynamic MTP rather than resting tongue pressure. Nevertheless, MTP serves as an effective surrogate for global functional reserve, with lower values indicating a reduced myofunctional capacity. This muscular weakness explains the structural inability to sustain a prolonged anti-gravity resting posture. Furthermore, MTP presents a distinct clinical advantage over complex electromyography (EMG) by providing a non-invasive, highly reproducible metric to evaluate myofunctional capacity.

To further evaluate this relationship, mediation analysis was applied to assess the pathways linking soft-tissue function and skeletal morphology. This statistical method evaluates whether an independent variable affects a dependent variable through a third mediating variable [32, 33]. The present analysis revealed a partial mediation pattern: while MTP maintained a significant direct association with the ANB angle, approximately one-third of its total influence was indirectly mediated by TTI. This dual pathway can be interpreted by integrating muscular capacity with the equilibrium theory of craniofacial morphogenesis [34]. While the direct pathway suggests that overall myofunctional capacity (MTP) is fundamentally linked to the skeletal base, the significant indirect pathway underscores the critical role of resting posture. According to the equilibrium theory, morphological adaptations are heavily influenced by the continuous, low-magnitude resting pressures exerted by the surrounding soft tissues. When reduced MTP cannot sustain an elevated resting posture, the tongue undergoes a compensatory anterior-inferior displacement, which is clinically reflected by a lower TTI. Under such conditions, the tongue tip maintains continuous resting contact against the lingual aspect of the mandible. This creates a sustained forward force that may promote mandibular protrusion, indirectly exacerbating the Class III skeletal discrepancy.

Notably, in both multivariable linear regression analysis and mediation analyses, TTI was the only postural variable significantly associated with the ANB angle. Nevertheless, vertical parameters such as TDD and TTD remain clinically relevant. Consistent with the volume conservation principle inherent to the muscular hydrostat, a low tongue posture involves a concomitant relationship between the sagittal protrusion of the tongue tip (decreased TTI) and the inferior drop at the tongue dorsum (increased TDD). Presumably, because the present study focuses primarily on sagittal skeletal alterations, TTI—which specifically reflects the sagittal spatial adaptation of the tongue—serves as a more sensitive and direct indicator in both predictive and mediating capacities compared to the vertical dimensions.

Traditional orthodontic diagnostics often rely on static cephalometry, which may not fully integrate morphological and dynamic functional factors. To address this, a CHAID decision tree algorithm was employed. Unlike traditional regression or opaque “black-box” machine learning models, decision trees are highly valued for their exceptional interpretability and intuitive visual structure [35]. As medical artificial intelligence increasingly shifts towards Explainable AI (XAI) to foster clinical trust [36], the CHAID model provides clinicians with a straightforward, rule-based framework by automatically partitioning data into mutually exclusive subgroups. The model identified MTP as the primary root node, indicating that reduced MTP (≤ 1.85 kg) is the principal functional indicator associated with skeletal Class III malocclusion. Subjects with a higher MTP (> 1.85 kg) were predominantly classified into Class I or II skeletal relationships. However, for patients exhibiting initial muscular weakness (MTP ≤ 1.85 kg), functional data alone were insufficient for precise stratification. In this subset, the model utilized TTI as a crucial secondary node. Within this functionally susceptible group, subjects with a severe anterior tongue position (TTI ≤ 2.57%) showed the highest probability of presenting a Class III pattern, whereas an elevated posture (TTI > 15.15%) corresponded to a significantly lower risk profile.

Clinically, the CHAID model translates these complex morpho-functional interactions into a potential sequential screening pathway. According to the observed statistical associations, the primary diagnostic tier necessitates the evaluation of muscular capacity, identifying an MTP ≤ 1.85 kg as the critical threshold for the Class III phenotype. For patients falling within this functionally vulnerable range, purely muscular parameters require further spatial contextualization. In this secondary diagnostic tier, the model highlights resting posture, identifying severe sagittal anterior displacement of the tongue tip (TTI ≤ 2.57%) as an indicator associated with the highest likelihood of Class III malocclusion.

These findings propose a theoretical two-step screening framework: initial morphological assessment (TTI) followed by myofunctional testing (MTP) for intermediate cases. While this multidimensional evaluation may conceptually aid in identifying candidates for early interceptive therapies, the cross-sectional design of the present study precludes drawing direct clinical conclusions regarding treatment efficacy. Instead, the observed mediation relationship generates a hypothesis for future interventional studies: managing skeletal Class III malocclusion associated with tongue dysfunction might require addressing both muscle strength and posture synergistically. It is hypothesized that interventions relying solely on spatial reminders without addressing muscle tone, or conversely, increasing muscle strength without spatial guidance, might yield limited long-term stability. Therefore, future longitudinal interventional studies are warranted to test the hypothesis that integrating resistance-based training (to increase MTP) with spatial exercises (for postural correction) within myofunctional therapy (MFT) protocols can effectively influence the skeletal base.

This study has several limitations. First, the cross-sectional design restricts our ability to determine definitive causality or temporal sequences among muscle strength, posture, and skeletal morphology. Although the mediation analysis demonstrates robust statistical associations, it reflects observations at a single time point rather than a proven developmental timeline. Longitudinal prospective studies are needed to validate the directionality of these relationships. Second, as an observational study, it lacks longitudinal interventional data. Therefore, the proposed clinical integration of tongue resistance training and postural correction for Class III management serves as a hypothesis for future research rather than a proven therapeutic protocol; randomized controlled trials are required to verify its actual clinical efficacy. Third, the CHAID model thresholds were derived from a localized sample of 182 subjects; multi-center validation is required to confirm their generalizability. Furthermore, we acknowledge that important potential confounding factors, such as body mass index (BMI) and three-dimensional volumetric airway status, were not quantitatively evaluated in the current dataset, which warrants further investigation in future studies.

## Conclusions

This study indicates that skeletal Class III malocclusion in adolescents is associated with reduced tongue pressure and a lowered resting posture. According to the mediation analysis, while tongue muscle force directly associates with the sagittal skeletal base, TTI mediates roughly one-third of this relationship. Furthermore, the diagnostic model highlights reduced MTP as the primary risk indicator for Class III patterns, which is further stratified by TTI.

## Data Availability

The datasets used and analyzed during the current study are available from the corresponding author on reasonable request.

## Abbreviations

MTP: Maximum active tongue pressure
TTI: Tongue tip index
TTD: Tongue tip distance
TDD: Tongue dorsum distance
